# Prevalence of Substance Use and Sleep-Related Problems Among Driver Victims Involved in Road Traffic Accidents Presenting to a Tertiary Care Hospital in India

**DOI:** 10.7759/cureus.74934

**Published:** 2024-12-01

**Authors:** Esther Lalringzo, Vishal Dhiman, Ravi Gupta, Bhaskar Sarkar, Ashish R Bhute, Manisha Naithani, Aniruddha Basu

**Affiliations:** 1 Department of Psychiatry, All India Institute of Medical Sciences, Rishikesh, Rishikesh, IND; 2 Department of Trauma and Orthopaedics, All India Institute of Medical Sciences, Rishikesh, Rishikesh, IND; 3 Department of Forensic Medicine and Toxicology, All India Institute of Medical Sciences, Rishikesh, Rishikesh, IND; 4 Department of Biochemistry, All India Institute of Medical Sciences, Rishikesh, Rishikesh, IND; 5 Department of Psychiatry, All India Institute of Medical Sciences, Kalyani, Kalyani, IND

**Keywords:** drivers, injury, road traffic accidents, sleepiness, substance use

## Abstract

Background: Road traffic accidents (RTAs) are a critical public health problem leading to significant morbidity, mortality, and socioeconomic losses. Despite known risk factors like substance use and sleep-related problems, there is limited research on the prevalence of these factors among drivers who met with RTAs. Hence, this study aimed to gain insight into the prevalence of substance use and sleep-related problems among this population attending a trauma center in the northern State of India.

Methodology: A cross-sectional study was conducted among 383 driver victims (DVs) who presented to a publicly funded tertiary care hospital's trauma emergency department of the Himalayan State of India following RTAs. The hospital's catchment area is vast and caters to people from both hilly and plain areas. Data were collected for sociodemographic characteristics, clinical parameters, and accident-related factors using a semi-structured proforma. Substance use-related problems were assessed through detailed history evaluation, thorough examinations, structured questionnaires, and body fluid (blood and urine) drug analysis. Sleep-related parameters were evaluated in detail, including excessive daytime sleepiness (EDS), the functional outcome of sleepiness, and the chronotype using structured and validated questionnaires. The nature, site of injuries, and their severity were determined using the Abbreviated Injury Severity (AIS) Scale.

Results: Among DVs, 221 (57.7%) tested positive for alcohol; 71 (18.6%) had used other psychotropic substances, with cannabis being the most common among them; and 56 (14.6%) reported using multiple substances. Eighty-three (21.7%) participants had EDS, and 102 (26.6%) experienced fatigue and sleepiness during the accident. The most common type of injuries was fracture and dislocation 206 (53.8%), with the extremities (both upper and lower) being the most common body region (218, 56.9%) involved, along with head traumas in equal proportions. Injuries were predominantly minor, yet a concerning 7.6% of the participants experienced severe trauma.

Conclusion: The study highlights the substantial role of substance use and sleep-related problems in RTAs, emphasizing the need for interventions targeting these factors to reduce the burden of RTAs. Policies enforcing stricter substance use regulations and promoting sleep health awareness and sleep assessments for drivers could significantly mitigate RTAs and improve road safety in India.

## Introduction

Road traffic accidents (RTAs) are a significant public health problem, representing approximately 25% of all injury-related deaths across the globe [1,2]. The World Health Organization (WHO) defines an RTA as “a road traffic event where there is a collision involving at least one moving vehicle, happening by chance or without an apparent or deliberate cause leading to injury which is fatal or non-fatal” [3]. According to the report of the WHO, RTAs account for over 1.35 million deaths and more than 50 million injuries in a year. In contrast to high-income countries, the risk of mortality is threefold higher in lower-middle-income countries (LMICs), although these countries have less than 1% of all motor vehicles, and approximately 92% of deaths due to RTAs occur in LMICs [1]. For every accident-related injury, many survivors faced short-term or permanent disabilities, leading to limitations in physical functioning, psychological challenges, and reduced quality of life. They may lead to substantial financial difficulties because of the long-term burden of care, medical costs, and lost wages.

India is a rapidly growing nation with one of the largest road networks, and road transport is the country's most common mode of transportation. The number of injuries and fatalities in RTAs has remained consistently high, accounting for 11% of accident-related deaths globally, which is about 20 times that of developed nations [4,5]. As per the "Road Accidents in India 2022" report by the Ministry of Road Transport and Highway (MoRT&H), Government of India (GOI), more than 4.5 lakh RTAs were reported, resulting in an equal number of injuries (443,366) and nearly 1.7 lakh mortalities [4]. The National Crimes Record Bureau (NCRB), Ministry of Home Affairs (GOI), in its latest report, illustrated similar data of 446,768 road accidents, 423,158 injuries, and 171,100 deaths [6]. Compared to neighboring states with similar vehicular density, India has significantly higher RTAs and injuries. Bangladesh and Nepal, two nearby nations, show similarly alarming patterns [7,8].

RTAs result from a multiple and complex interplay of human errors, road infrastructure, and environment and vehicular conditions [5]. However, human (driver)-related factors are the most significant, accounting for about 78% of accidents [9]. Substance use, including alcohol and other psychotropic substances, is a widely recognized considerable risk factor that significantly inflates the risk of RTAs to two- to sixfold, and multiple substance use poses a greater risk [10-14]. Substance use impairs driving by slowing reaction time, blurring vision, fostering false confidence, diminishing concentration, and encouraging aggressive driving [12]. In the year 2022, 2.2% of RTAs in India were due to consumption of alcohol and any other drug(s)[4]. MoRT&H, GOI, reported that among all road accidents, 2.2% was the share for drunken driving or consumption of alcohol and drugs, whereas it caused 2.5% of all fatalities [4].

Furthermore, sleepiness is the next leading risk factor for RTAs, attributing to around 10% to 30% of fatal accidents and 42.5% of near-miss accidents [13,14]. The literature suggests that sleepiness is influenced by fatigue, sleep deprivation, and other sleep disorders like insomnia, obstructive sleep apnea, and sleep work disorder [15,16]. Sleepiness, characterized by difficulty in maintaining wakefulness or alertness, is suggested to impair cognitive functioning and increase reaction time leading to a higher risk of accidents [17,18]. Chronotype, which is referred to as the tendency of a person to go to sleep at a specific hour [19], is another factor affecting driving performance, where people with an evening chronotype are more prone to circadian misalignment and have a 15.2 times higher likelihood of experiencing drowsy driving as compared to morning types [20]. Moreover, drivers who sleep for less than five hours face nearly five times the risk of being involved in an RTA due to impaired vigilance and concentration [21].

A review of the literature suggests that most Indian studies have acknowledged substance use as a contributor to road accidents. However, they have primarily utilized a retrospective approach to report it [5,22-24]. There needs to be more data on the use of other psychoactive substances as a contributor to RTAs [22,25,26]. In addition, there is a significant gap in epidemiological data on the prevalence of sleep-related problems among driver victims (DVs). India's central databases on RTAs, i.e., NCRB and the MoRT&H Reports, notably overlook sleepiness, other sleep disorders, and drugs other than alcohol as factors contributing to road accidents [4,6]. To bridge these gaps, the primary aim of our study was to assess the proportion, type, and pattern of substance use among DVs. The aim goes beyond the traditional focus on alcohol and investigates the role of other psychotropic substances among drivers involved in a road accident. The study also examines the prevalence of excessive daytime sleepiness (EDS) among these drivers. Unlike alcohol-related accidents, there are no simple tools to measure sleepiness and fatigue levels, leading to a lack of data on the impact of sleepiness on road safety. Hence, the current study's findings may provide some better insights in this regard.

Part of the findings of this article was presented as an oral presentation during the 18th Uttarakhand State Science and Technology Congress-2024, which was held on February 8-9, 2024, at Uttarakhand Open University, Haldwani, Uttarakhand, India. This presentation won the Young Scientist Award at the mentioned conference.

## Materials and methods

**Figure 1:**
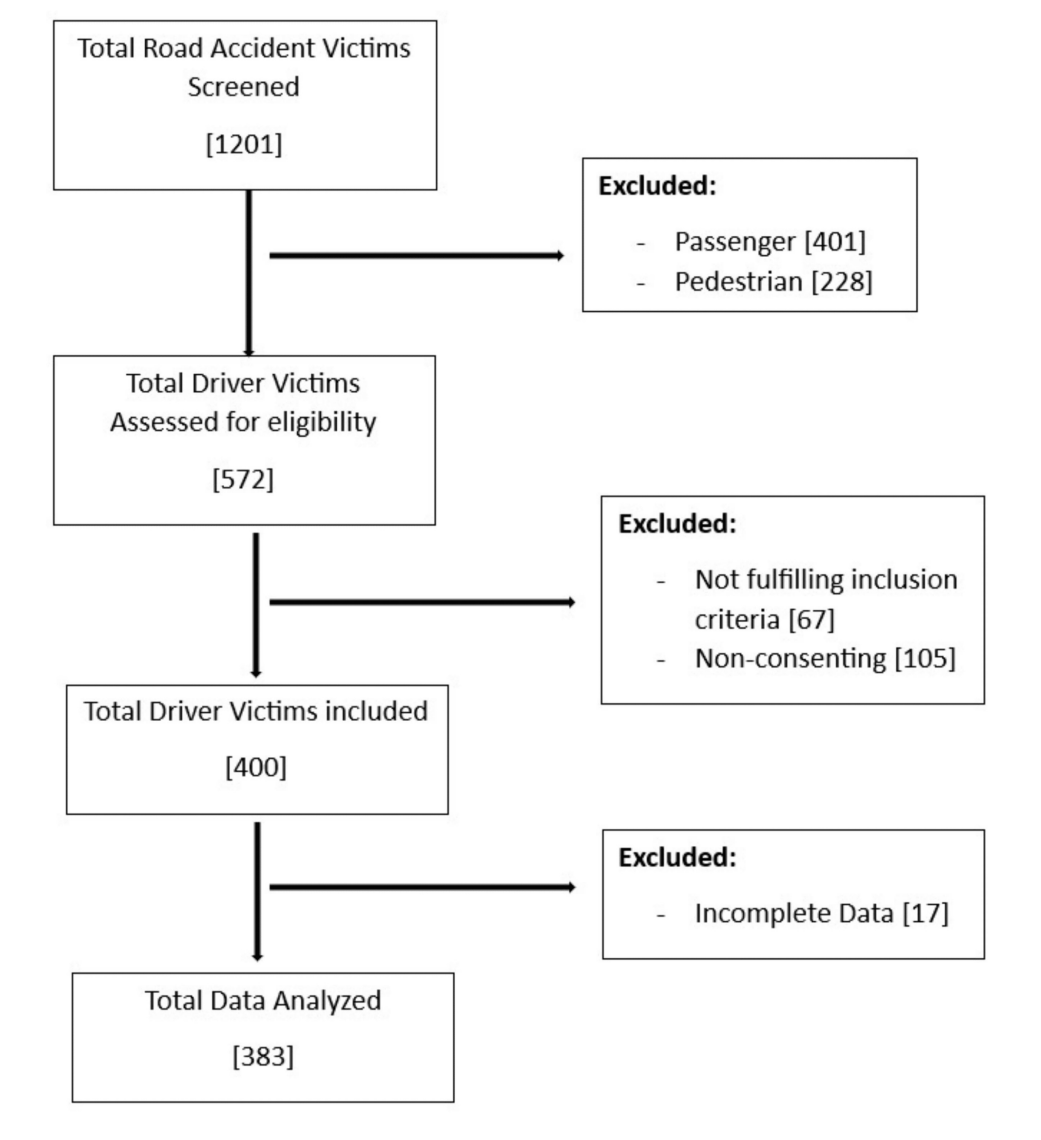
Consort diagram of participant recruitment

This was a single-center, cross-sectional study conducted among DVs of RTAs presenting to the Accident and Emergency (A&E) Care Unit, Department of Trauma Surgery and Critical Unit of All India Institute of Medical Sciences, Rishikesh, a publicly funded teaching-training medical school in northern India. A convenient sampling technique was used to enroll the participants, i.e., DVs attending A&E due to vehicular RTAs. A total of 1,201 DVs were screened using inclusion and exclusion criteria, and 383 consenting DVs between 18 and 60 years were enrolled in the study. Those who presented beyond 48 hours of RTAs were excluded from the study. After explaining the rationale of the study, written informed consent was obtained from the participants or their primary caregivers. The flow of participants in the study is illustrated in Figure 1.

A semi-structured Performa was used to collect baseline sociodemographic details and other clinical parameters, such as driving experience, history of previous RTA, injury in the last RTA, history of traffic challans, and legal issues related to road traffic rules. The proforma also included information on road infrastructure collected, such as material (concrete/earthen), type (highway/non-highway), a straight or non-straight road, and the type of vehicle involved in the accident. In addition, information about the use of safety equipment (helmet or seatbelt), the kind of collision, the time of the accident, and if there were any road traffic rule violations were also recorded. About 5 ml of venous blood and a urine sample (10 ml in a sterile container) were collected immediately (within two hours of admission) and sent to the biochemistry laboratory for further processing per the protocol. The Institutional Ethics Committee of All India Institute of Medical Sciences, Rishikesh, approved the present study protocol (approval no. AIIMS/IEC/20/731). All collected information obtained from the participants was anonymized.

Sample size

Based on a previous study [27], considering the proportion of outcome variable as 50% in the study population, with 5% precision and 95% confidence interval, the calculated sample size was 381.

Measures

Substance Use Assessment

The self-reported Alcohol, Smoking, and Substance Involvement Screening Test (ASSIST) was used to assess the risk of addiction to various substances: tobacco, alcohol, cannabis, cocaine, amphetamine-type stimulants, sedatives, hallucinogens, inhalants, opioids, and other drugs. This questionnaire consists of eight questions, and the total score differentiates use, abuse, and dependence on various addictive substances. The test has good internal consistency [28].

Blood Alcohol Concentration (BAC) Analysis

The blood sample was centrifuged at 4,000 revolutions per minute for 10 minutes. The serum was separated and stored at -800 °C until laboratory analysis. The blood alcohol concentration (BAC) was determined using the Beckman Coulter Chemistry Analyzer AU480 instrument. This method exclusively detects ethyl alcohol and can accurately quantify alcohol concentration in the blood sample containing 0.01-0.60% alcohol. This method is based on the alcohol dehydrogenase principle, where the enzyme alcohol dehydrogenase oxidizes the blood alcohol to acetaldehyde. The measured absorbance at 376 nm due to the production of NADH+ (nicotinamide adenine dinucleotide hydrogen) from NAD+ is proportional to the alcohol concentration in the blood sample.

Urine Drug Screen

It was conducted using a six-panel urine drug kit test that detects drugs such as amphetamines, barbiturates, benzodiazepines, cannabis, cocaine, and morphine. The urine sample was collected within two hours of the patient's arrival at the Trauma Emergency Department. This rapid test works on the principle of specific immunochemical reactions between antibodies and antigens to identify the particular compounds in the urine sample. The assay depends on the competitive binding between the drug conjugate in the test kit and the free drug potentially present in the urine sample.

Morningness-Eveningness Questionnaire (MEQ)

The Morningness-Eveningness Questionnaire (MEV) is widely used to assess individual differences in circadian rhythms [29]. The questionnaire demonstrated internal solid reliability, with scores ranging from 0.70 to 0.86 and high stability measured by Cronbach's alpha ranging from 0.84 to 0.95. The questionnaire consists of 19 questions and categorizes individuals as morning, intermediate, and evening types based on total scores ranging from 16 to 86. It is simple to understand, easy to administer, and reliable to assess chronotype.

Epworth Sleepiness Scale (ESS)

The Epworth Sleepiness Scale is the most widely used questionnaire for evaluating EDS [30]. It is brief, easy to understand and administer, assessing the likelihood of dozing off during eight daily situations. The ESS has strong internal consistency with a Cronbach's alpha of 0.73 to 0.88 and high test-retest reliability [31]. Analyzing the item responses indicates that the ESS effectively measures sleepiness [32]. The questionnaire accurately differentiates individuals with and without EDS based on the total score, which ranges from 0 to 24, with scores above 10 suggesting EDS [30].

Functional Outcome of Sleep Questionnaire (FOSQ-10)

The Functional Outcome of Sleep Questionnaire (FOSQ-10) is used to evaluate how excessive sleepiness affects everyday activities. It demonstrates high internal validity with a Cronbach’s alpha of 0.87 and strong test-retest reliability (r = 0.96) [33]. The questions are simple and can effectively distinguish between normal individuals without sleepiness-related impairments and those experiencing daily limitations due to excessive daytime sleepiness. The scoring system ranges from 5 to 40, with each sub-scale’s average score combined to produce a total score between 5 and 40, where a higher score reflects a better functional outcome.

Abbreviated Injury Scale (AIS)

The AIS is a commonly used scoring system to assess injury severity and predict outcomes in patients with multiple injuries [34]. It has demonstrated high reliability and consistent scores when evaluating the same patient at different times. The AIS effectively predicts the necessary treatment required, with scores ranging from 0 to 75; a score of 16 or higher indicates major trauma, while a score above 40 signifies massive trauma.

Statistical analysis

Analysis was done using IBM SPSS Statistics for Windows, version 29.0 (released 2023, IBM Corp., Armonk, NY). Categorical variables were summarized as frequencies/percentages and continuous variables as mean ± SD.

## Results

Three hundred eighty-three participants were included in the study, where 343 (89.6%) were males and the rest were female. The mean age was 31.41 years (SD ±9.76), and the average body mass index (BMI) was 23.19 kg/m^2^ (SD ±2.91). Educationally, 204 (53.3%) had completed up to the 12th standard, and the majority (230, 60.1%) were employed. Nearly half of the participants (182, 47.5%), belonged to the lower socioeconomic strata. Only 48 (12.5%) of the participants were professional drivers. The average working duration of the participants was 8.32 ± 2.40 hours, and approximately one-fourth (89, 23.2%) were working on a shift basis (Table 1). 

**Table 1:** Sociodemographic details of the participants

Variable	Category	n	%
Gender	Male	343	89.6
	Female	40	10.4
Age Group	18-30	216	56.4
	31-45	134	35
	46-60	33	8.6
Marital Status	Single	178	46.5
	Married	203	53
	Others	3	0.7
Education	Illiterate	13	3.4
	Primary	67	17.5
	High	99	25.8
	Secondary	131	34.2
	Graduate and above	73	19.1
Occupation	Employed	230	60.1
	Unemployed	94	24.5
	Student	59	15.4
Socioeconomic status	Upper	60	15.7
	Middle	141	36.8
	Lower	182	47.5
BMI category	Underweight	11	2.9
	Normal weight	183	47.8
	Overweight	189	49.3
Driver type	Non-professional	335	87.5
	Professional	48	12.5
Shift work	Yes	89	23.2
	No	294	76.8
Work duration (hours)	7 hours	139	36.3
	8-10 hours	199	52
	>10 hours	45	11.7

**Figure 2:**
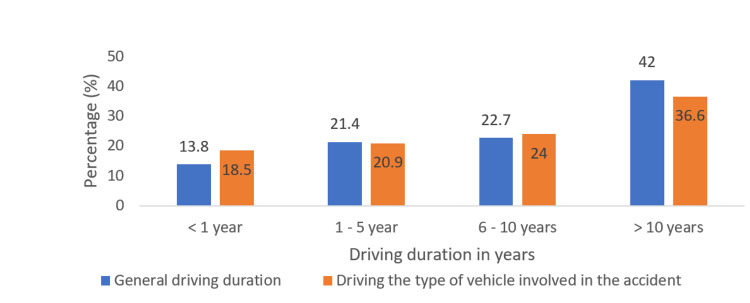
Driving experience of the participants

The participants had an average driving duration of 11.46 ± 9.06 years, with the specific driving experience of the vehicle involved in the accident being 10.62 ± 8.9 years (Figure 2).

**Figure 3:**
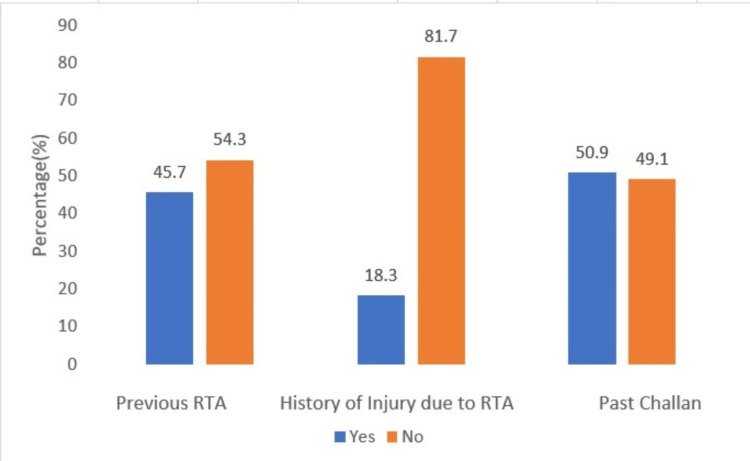
Details on previous involvement in road traffic accident (RTA), injury sustained in the previous accident, and history of challan

One hundred seventy-five (45.7%) participants reported prior involvement in a road accident, while 70 (18.3%) indicated they had sustained injuries from these accidents. In addition, 195 (50.9%) had previously received a traffic citation (challan tickets) (Figure 3).

The majority, 273 (71.3%), held a valid driver's license, and 289 (75.5%) were driving a two-wheeler at the time of the accident. A slip or loss of control (128, 33.4%) and a hit from the back (90, 23.5%) were the two most frequently reported causes of accidents. Over half of the accidents, 203 (53.0%), occurred on the highway, with 242 (63.2%) taking place on straight roads and 257 (67.1%) on concrete surfaces. Weekdays accounted for 208 (54.3%) of the accidents, while the remaining incidents occurred on weekends. The peak accident time was between 6:00 PM and 12:00 midnight (144, 37.6%). Most accidents, 222 (58.0%), occurred under sufficient lighting conditions, although some occurred in foggy or dark conditions (Table 2).

**Table 2:** Distribution of accident characteristics by vehicle, collision, and road and environmental factors

Variable	Category	n	%
License type	No license	94	24.5
	Valid license	273	71.3
	Learners license	12	3.1
	Expired license	4	1.0
Vehicle type	2-wheeler	298	75.5
	4-wheeler	78	20.4
	Others	16	4.2
Collision type	Head-on	83	21.7
	Hit from the back	90	23.5
	Side impact	59	15.4
	Vehicle overturn/run off-road	23	6
	Slip/loss of control	128	33.4
Road type	Highway	203	53
	Non-highway	180	47
Road material	Earthen	55	14.4
	Concrete	257	67.1
	Others	71	18.5
Road features	Straight	242	63.2
	Others	141	36.8
Day of accident	Weekdays	208	54.3
	Weekends	175	45.7
Time of accident	6 AM to 12 noon	72	18.8
	12 noon to 6 PM	105	27.4
	6 PM to 12 midnight	144	37.6
	12 midnight to 6 AM	62	16.2
Lighting condition	Sufficient light	222	58
	Foggy	22	5.7
	Darkness	139	36.3
Road familiarity	Yes	259	67.6
	No	124	32.4

**Figure 4:**
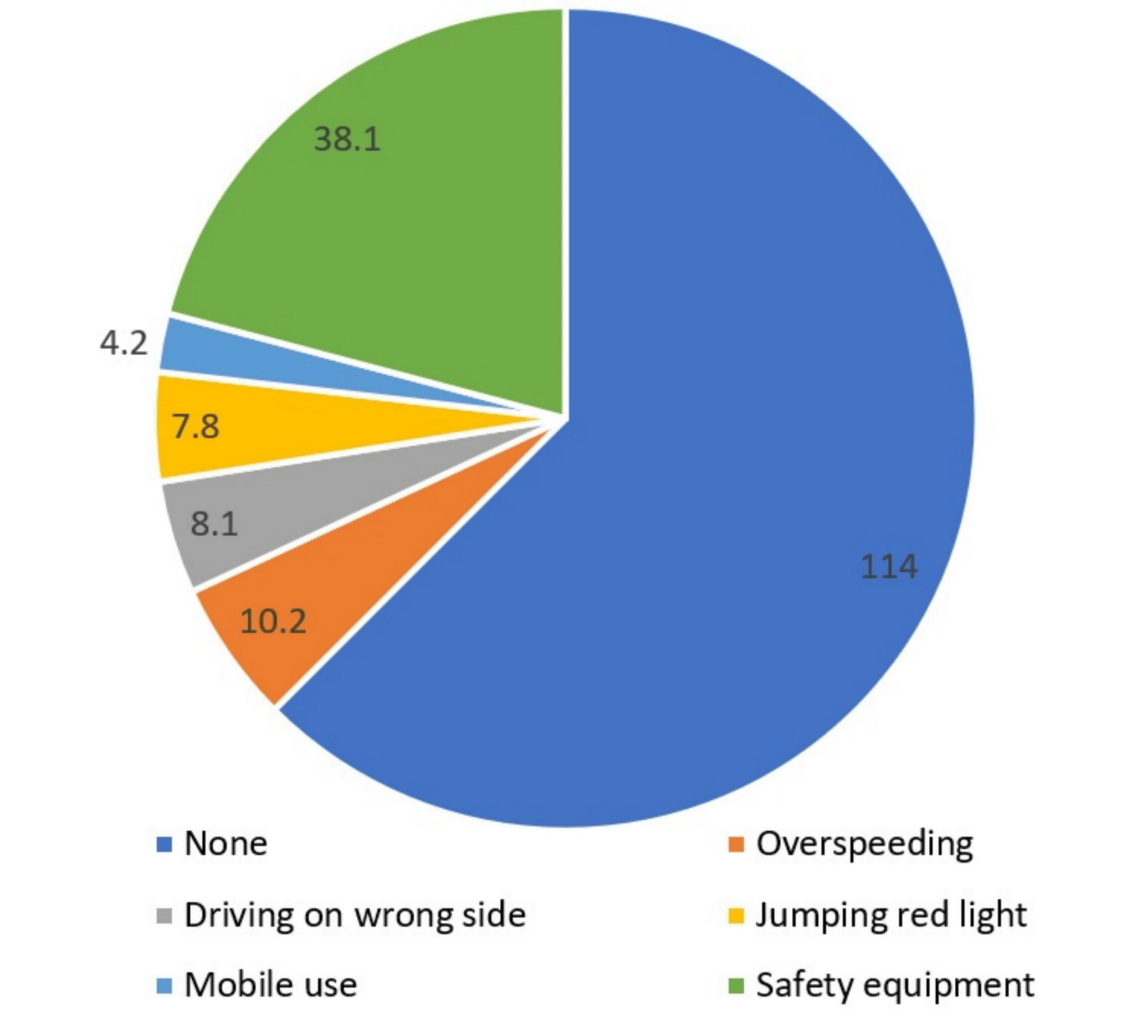
Traffic rules violated at the time of the accident

Among the traffic violations reported, driving without a helmet or seatbelt was the most prevalent (146, 38.1%), followed by overspeeding (Figure 4).

The average sleep duration the night before the accident was 7.27 ±2.04 hours. Eighty-three (21.7%) of the participants had an ESS score of greater than 10, indicating the presence of EDS. Ironically, the majority, 305 (79.6%), of the participants were found to be a morning type of person (Table 3).

**Table 3:** Distribution of participants based on sleep-related factors, daytime sleepiness, and chronotype

Variable	Category	n	%
Sleep duration (previous night)	<5 hours	32	8.4
	5-8 hours	245	64
	>8 hours	106	27.7
Countermeasures for sleep	Tea	47	12.3
	Coffee	40	10.4
	Others	16	4.2
Excessive daytime sleepiness	Yes	83	21.7
	No	300	78.3
Chronotype	Morning	305	79.6
	Neither	77	20.1
	Evening	1	0.3

**Figure 5:**
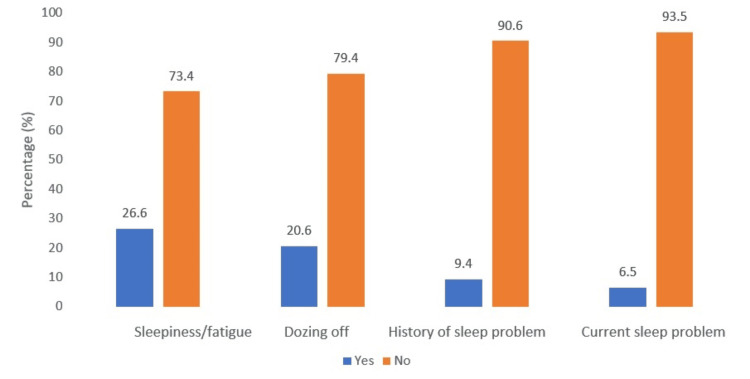
Sleepiness and its related details of the participants

Among the participants, 102 (26.6%) reported experiencing sleepiness or fatigue while driving at the time of the accident, and 79 (20.6%) reported dozing off while driving. A minority, 36 (9.4%), had a history of sleep disorders, and 25 (6.5%) reported sleep problems at present (Figure 5).

The FOSQ table indicates that participants reported moderate to high levels of general productivity (3.64 ± 0.49), activity level (3.85 ± 0.26), and vigilance (3.72 ± 0.32). Social outcomes were also moderately high (3.67 ± 0.53). However, intimacy (2.22 ± 1.98) indicates a comparatively less engagement (Table 4).

**Table 4:** Functional outcome of the sleep details of the participants

Functional Outcome of Sleep (FOSQ)		Mean ± SD
Sub-scales	General productivity	3.64 ± 0.49
	Activity level	3.85 ± 0.26
	Vigilance	3.72 ± 0.32
	Social outcome	3.67 ± 0.53
	Intimacy	2.22 ± 1.98

A total of 126 (32.9%) participants self-reported driving under the influence of substances at the time of the accident; however, objective assessments revealed that 221 (57.7%) were under the influence of alcohol, 71 (18.6%) had used psychotropic substances (8.4% on cannabis, 6.8% on benzodiazepines, 0.5% on cocaine, and 2.9% on opioids), and 56 (14.6%) were found to be using multiple substances (n = 56; 14.6%). The average blood alcohol concentration was 1.00 ± 1.01 g/l, with cannabis being the most frequently detected drug use among the drivers (Table 5).

**Table 5:** Distribution of participants on the basis of substance use and its type

Variable	Category	n	%
Driving under the influence (self-reported)	Yes	126	32.9
	No	257	67.1
History of DUI	Yes	73	19.1
	No	310	80.9
Substance use problem (self-diagnosed)	Yes	63	16.4
	No	320	83.6
Blood alcohol concentration (BAC)	0.3-1.2 g/l	68	17.8
	1.2-2.5 g/l	126	32.9
	>2.5 g/l	27	7
Urine drug test results	Benzodiazepine	26	6.8
	Cocaine	2	0.5
	Opioids	11	2.9
	Cannabis	32	8.4
Poly substance use	Yes	56	14.6
	No	179	46.7

**Figure 6:**
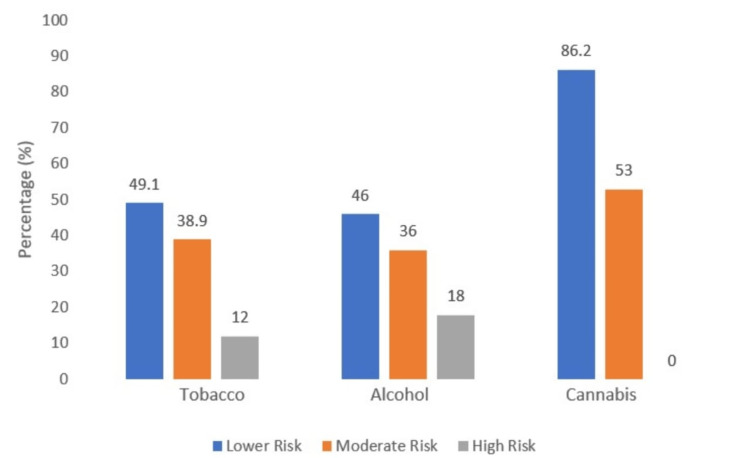
Risk of substance use according to the Alcohol, Smoking and Substance Involvement Screening Test (ASSIST)

According to the ASSIST scores, 69 (18%) participants were classified as high-risk for alcohol use. In comparison, 53 (13.8%) were at moderate risk for cannabis use (Figure 6).

The average time to reach the hospital following the accident was 2.86 ± 2.64 hours, with a majority (251, 65.5%) receiving some form of medical assistance before hospital admission. Fractures or dislocations were the most sustained injuries, 206 (53.8%), predominantly affecting the extremities (upper and lower limbs). Of the many participants, 240 (62.7%) sustained only minor injuries, and a notable 29 (7.6%) suffered severe injuries because of the accident (Table 6).

**Table 6:** Distribution of participants based on the injury sustained due to the accident

Category	Subcategory	n	(%)
Time taken to reached hospital	Within 1 hour	185	48.3
	2-10 hours	189	49.3
	>10 hours	9	2.3
Types of injuries	Soft tissue injury	125	32.6
	Fracture/dislocation	206	53.8
	Head injury	74	19.3
	Others	70	18.3
Medical assistance	Yes	251	65.5
	No	132	34.5
Injured body regions	Head region	217	56.7
	Trunk region	155	40.5
	Extremities	218	56.9
Severity of injury	Minor	240	62.7
	Moderate	114	29.8
	Severe	29	7.6

## Discussion

The data from this study offer valuable insights into the sociodemographic, behavioral, and situational factors associated with RTAs among drivers in India, alongside substance use and sleepiness-related impairments impacting driver safety. The findings reveal that a substantial proportion of drivers involved in RTAs were young males (89.6%), predominantly aged 18-30 years, which aligns with national central databases [4,6]. Furthermore, similar trends have been reported by Pathak and colleagues in their research from Pune, and the reported majority were young (20-30 years) males (84.6%) [35]. Similarly, Roy et al., in their study from the neighboring state of Bangladesh, observed similar findings (males, 76.0%, mostly between 20 and 35 years of age (42.90%)) [36]. This consistent pattern may be attributed to the likelihood that males, particularly younger, are more likely to engage in risky driving behaviors such as overspeeding, rash driving, and disobeying traffic rules [37].

Notably, two-wheeler drivers represented the largest vehicle group involved in RTAs (75.5%), which aligns with the findings nationwide [4,6]. Pathak et al. [35] reported that motorized two-wheelers were the most common (71.9%) type along with another study by Sangal et al., who had a similar finding of 82.52% [38]. This finding may be secondary to the fact that the number of vehicles on the road for two-wheelers may be higher than others (three- and four-wheeler vehicles) and may be confirmed from the national sales and Regional Transport Offices’ records.

In our study, 38.1% of participants failed to use helmets or seatbelts at the time of the accident. The latest annual report published by the Ministry of Road Transport and Highways (MoRTH) has reported that a high prevalence of drivers who were injured or killed due to a road accident did not wear safety equipment (helmet/seatbelt) at the time of the accident [3]. A Yemen-based study conducted by Alfalahi et al. reported that none of the RTA victims wore seatbelts and helmets [39], which is consistent with our study findings. Our study participants reported that safety equipment like seatbelts and helmets are mostly considered uncomfortable, especially during hot and humid weather.

Slip or loss of control was the most reported (33.4%) cause of an accident, often occurring on highways (53%) and straight roads (63.2%) with concrete surfaces [1,40]. Such conditions can exacerbate the chances of accidents while overspeeding and limited driver control, further compounded by nighttime driving, accounting for 37.6% of the RTAs, which aligns with other research findings [23,41]. Our study reveals that concrete roads and weekend driving during nighttime are accident-prone factors, calling for targeted measures to improve infrastructure and enhance road safety during these high-risk periods. To address this multifactorial issue effectively, strategies should incorporate measures from a public health perspective, including stringent enforcement, early interventions, and road safety campaigns tailored to at-risk demographics.

Despite the documented inter-relationship between substance use and the risk of accidents, many drivers continue to drive under the influence of substances, which is probably underreported due to limited testing [42,43]. The current study showed that 57.7% of the drivers had used alcohol before RTAs, supporting the results of similar analyses worldwide [22,27,38,40,44,45]. We found that 18.6% of DVS were under the influence of other psychotropic substances also, like cannabis (8.4%), which was the next most common drug of use, followed by benzodiazepines (6.8%), cocaine (0.5%), and opioids (2.9%). Among those found positive for substance use, 14.6% (56%) used it in a polysubstance use pattern. Our findings align closely with other national and international studies, which reported a similar proportion of substance use among drivers involved in RTAs [22,44,45]. Furthermore, a national survey report on the magnitude of substance use in India reported that cannabis is the second most common type of substance use in India, next to alcohol [46].

In our study, seep-related problems have emerged as a critical factor in relation to RTAs. A percentage of 26.6% of DVs experienced fatigue and sleepiness while driving and 20.6% reported dozing off at the wheel. These findings are similar to earlier literature, which suggests that most of the DVs doze off at the wheel secondary to insufficient nighttime sleep [18,21] and EDS in 21.7% (n = 83), which may be measured using the ESS [21]. It is recommended that a substantial proportion of drivers who experience EDS need interventions to address sleep hygiene and sleep healthcare education. Our study reports that 37.6% of DVs had taken inadequate rests, and the finding is in support from earlier similar studies [20,47]. The chronotype of most of the participants was identified as morning type (n = 305; 79.6%), and they met accidents during the evening and late day hours. It indicates a strong possibility of circadian misalignment, which could have contributed to fatigue, drowsiness, dozing off at wheels, and attentional deficits. This finding of ours is in alignment to previous research conducted on a similar population and has suggested that evening-oriented individuals are more vulnerable to accidents [48].

In terms of injury patterns, the majority of the participants (n = 206; 53.8%) sustained fractures and dislocations, with extremities (n = 218; 56.9%) and head (n = 217; 56.7%) traumas being the most frequently injured body regions. Injuries were predominantly minor, yet a concerning 7.6% (n = 29) of participants experienced severe injuries, underscoring the need for prompt medical intervention. This injury pattern is consistent with findings from other studies conducted in different countries [36,39,49,50]. The average delay in reaching the hospital was approximately 2.9 hours from plain areas, whereas it took a few hours to more than three to five hours in cases of events occurring in hilly areas.

The current research examined sociodemographic parameters and substance use patterns, unlike other studies. It dived into assessing the magnitude of sleep-related problems among DVs of RTAs presenting to a trauma center situated in the foothills of a northern Himalayan State of India, providing a better understanding of the epidemiology and distribution of multiple associative factors of road accidents. Understanding these contributing factors can help the authorities and policymakers develop and improve national and regional policies. Our study is one of the earlier studies for the mapping of multiple factors, including drugs (other than alcohol), chronotype, and varied sleep disorders. Although the current study provides a deeper insight into the role of substance use and sleep-related factors in road safety, several limitations must be acknowledged. First, the reliance on self-reporting certain variables, such as sleepiness on the wheel, dozing off while driving, may introduce recall and reporting biases, potentially impacting data accuracy. In addition, the study was conducted in a single hospital setting, which may limit the generalizability of the findings to broader populations. Further research across multiple regions and employing longitudinal study designs could provide more comprehensive insights and better guide the development of targeted public health interventions.

## Conclusions

From a humanitarian standpoint, it is imperative to mitigate injuries and deaths due to road accidents, especially in developing countries like India. This study, therefore, offers essential insights into the prevalence and types of substance use as well as sleep-related factors among drivers involved in RTAs in India, highlighting significant public health concerns. Findings indicate that a considerable proportion of drivers were under the influence of alcohol or other psychotropic substances, particularly cannabis, and many had an inadequate sleep duration before the accident, suggesting that these factors play a critical role in road safety, with implications for increasing accident risk and severity. This will guide the authorities and policymakers on the need to strengthen the enforcement of the existing laws against driving under the influence of alcohol and undergoing regular roadside screenings for alcohol and other drugs wherever feasible. These findings also highlight the need for spreading more public awareness to educate the public about the importance of sleep and the risks associated with RTAs, as sleepiness is usually overlooked as a significant risk factor for RTAs.

Moreover, standardized laws such as regular screenings for sleep problems should be mandated among license holders, and strict punishments should be imposed on people failing to do this. Lastly, more comprehensive research in collaboration with different related fields and among larger groups is necessary for understanding the fundamental causes of RTAs, enabling the government and policymakers to implement new regulations or enhance existing road safety measures to mitigate road accidents and their burden.
